# Compensatory and additive helper effects in the cooperatively breeding Seychelles warbler (*Acrocephalus sechellensis*)

**DOI:** 10.1002/ece3.4982

**Published:** 2019-02-14

**Authors:** Lotte A. van Boheemen, Martijn Hammers, Sjouke A. Kingma, David S. Richardson, Terry Burke, Jan Komdeur, Hannah L. Dugdale

**Affiliations:** ^1^ School of Biological Sciences Monash University Clayton Victoria Australia; ^2^ Behavioural and Physiological Ecology, Groningen Institute for Evolutionary Life Sciences University of Groningen Groningen The Netherlands; ^3^ Behavioural Ecology Group, Department of Animal Science Wageningen University & Research Wageningen The Netherlands; ^4^ School of Biological Sciences University of East Anglia Norwich UK; ^5^ Nature Seychelles Mahé Republic of Seychelles; ^6^ Department of Animal and Plant Sciences University of Sheffield Sheffield UK; ^7^ Faculty of Biological Sciences, School of Biology University of Leeds Leeds UK

**Keywords:** additive care, compensatory care, cooperative breeding, investment strategies, load‐lightening, parental care, Seychelles warbler

## Abstract

In cooperatively breeding species, care provided by helpers may affect the dominant breeders’ investment trade‐offs between current and future reproduction. By negatively compensating for such additional care, breeders can reduce costs of reproduction and improve their own chances of survival. Alternatively, helper care can be additive to that of dominants, increasing the fledging fitness of the current brood. However, the influence helpers have on brood care may be affected by group size and territory quality. Therefore, the impact of helping needs to be disentangled from other factors determining offspring investment before conclusive inferences about the effect of help on additive and compensatory care can be made. We used 20 years of provisioning data to investigate the effect of helping on provisioning rates in the facultative cooperatively breeding Seychelles warbler *Acrocephalus sechellensis*. Our extensive dataset allowed us to statistically disentangle the effects of helper presence, living in larger groups and different food availability. We show compensatory and additive care (i.e., partial compensation) in response to helper provisioning. Helpers lightened the provisioning load of the dominant male and female and increased total provisioning to nestlings. This was irrespective of group size or territory quality (food availability). Moreover, our results illustrate sex‐specific variation in parental care over the course of the breeding event. We discriminate between temporal variation, group size, and territory quality processes affecting cooperative care and as such, gain further insight into the importance of these factors to the evolutionary maintenance of helping behavior.

## 1 INTRODUCTION

In cooperative breeding systems, offspring care is often shared between the dominant male and female “breeders,” and a variable number of subordinate helpers (Koenig & Dickinson, 2016; Komdeur et al., 2017; Solomon & French, 1997; Stacey & Koenig, 1990). The optimal amount of parental investment provided by a dominant breeder is determined by the trade‐off between current and future reproduction (Stearns, 1989, 1992; Williams, 1966) and the care provided by helpers may affect the balance of this trade‐off for the dominants (Johnstone, 2011; Russell, Young, Spong, Jordan, & Clutton‐Brock, 2007). For example, care provided by helpers may increase the success of the current reproductive attempt, allow the dominants to reproduce more frequently, and/or improve the survival and future reproductive output of the dominants (Brown, Dow, Brown, & Brown, 1978; Heinsohn, 2004; Kingma, Hall, Arriero, & Peters, 2010; Koenig & Dickinson, 2016).

The investment strategies implemented by cooperative breeders are generally classified as “additive” and “compensatory” care strategies (Hatchwell, 1999a; Johnstone, 2011). When helpers improve overall care levels, the care provided is additive (Emlen & Wrege, 1991; Tanaka, Frommen, Engqvist, & Kohda, 2017; Zöttl, Fischer, & Taborsky, 2013). The resulting increase in the total amount of care received by the offspring can lead to higher reproductive success (Bales, French, & Dietz, 2002; Emlen & Wrege, 1991; Hatchwell, 2004; Komdeur, 1994; Russell et al., 2007; Tanaka, Kohda, & Frommen, 2018) through accelerated offspring growth (Bell et al., 2014; Dickinson, Koenig, & Pitelka, 1996; Hodge, 2005) and reduced offspring starvation (Dickinson et al., 1996; Hatchwell, 1999b, 1999a, 2004; Heinsohn, 1995; Kingma et al., 2010). Conversely, when the dominants compensate for the care provided by helpers by reducing their amount of care, the total amount of care received by the offspring may remain similar. Such “load lightening” by helpers can reduce the costs of reproduction for the dominants (Bruintjes, Heg‐Bachar, & Heg, 2013; Dixit, English, & Lukas, 2017; Heinsohn, 2004; Koenig & Walters, 2011; Meade, Nam, Beckerman, & Hatchwell, 2010; Scantlebury, Russell, McIlrath, Speakman, & Clutton‐Brock, 2002; Sharp, English, & Clutton‐Brock, 2013), which can lead to increased dominant survival (Cockburn et al., 2008; Hatchwell & Russell1996a; Heinsohn, 1992; Khan & Walters, 2002; Kingma et al., 2010) and increased future reproductive success (Brown & Brown, 1981; Russell, Brotherton, McIlrath, Sharpe, & Clutton‐Brock, 2003; Woxvold & Magrath, 2005; Blackmore & Heinsohn, 2007; but see Meade et al., 2010).

These additive and compensatory investment strategies are not mutually exclusive (Hatchwell1999a; Kingma et al., 2010) and theory predicts the optimal stable solution is for parents to incompletely compensate for additive care (load lightening) provided by additional carers (partial compensation), resulting in an increase in care received by offspring (Lessells & McNamara, 2012). The degree of parental response may be driven by the likelihood of offspring starvation, with more additive care when the risk of offspring starvation is higher, and more compensatory care when the risk of starvation is lower (Hatchwell, 1999a; Johnstone, 2011; Savage, Russell, & Johnstone, 2012).

Load‐lightening and additive care strategies have been studied in many cooperative breeding systems (Hatchwell, 1999a; Hatchwell & Russell, 1996a; Heinsohn, 2004; Liebl, Nomano, Browning, & Russell, 2016; MacGregor & Cockburn, 2002; McDonald, Kazem, & Wright, 2009; Russell, Langmore, Gardner, & Kilner, 2008; Wright & Dingemanse, 1999), but it is often extremely difficult to disentangle the effect of helpers from the effects of living in a larger group or on different quality territories (Cockburn et al., 2008; Dickinson & Hatchwell, 2004; Kingma, Santema, Taborsky, & Komdeur, 2014). For example, larger groups with more helpers may be better able to occupy territories with higher food availability; hence, the level of care to offspring might increase as a consequence of higher food availability in territories with helpers and not because of the contribution of helpers per se. Similarly, if more individuals occupy the territory and utilize the food sources, apparent load lightening of breeders could instead be the consequence of their reduced provisioning when food is more difficult to find; in such cases breeders would not actually reduce the amount of energy they expend in providing care. However, studies on load‐lightening and additive care disentangling the impact of helping from that of living in a larger group or in a territory with higher food availability are rare (e.g., Liebl et al., 2016; Cockburn et al., 2008).

Here, we use 20 years of parental and group provisioning data to investigate how helpers affect both breeder and overall offspring provisioning rates in the facultative cooperatively breeding Seychelles warbler *Acrocephalus sechellensis*. Seychelles warblers live in groups that occupy stable territories that are defended year‐round (Komdeur, 1991). Groups consist of a pair‐bonded dominant male and female and 0–5 subordinate individuals of either sex that may or may not provide help with provisioning nestlings and fledglings (Kingma, Bebbington, Hammers, Richardson, & Komdeur, 2016; Komdeur, 1994). The presence of subordinate helpers and nonhelping subordinates provides the opportunity to disentangle the impact of helping and group size (Woxvold & Magrath, 2005). Subordinates are generally retained offspring from previous reproductive attempts in the territory (but see Richardson, Burke, & Komdeur, 2007; Groenewoud et al., 2018). Dominant individuals gain from helper care as this positively influences the first‐year survival of offspring (Komdeur, 1994), an effect that persists into the adulthood of offspring receiving additional care (Brouwer, Richardson, & Komdeur, 2012). A previous study on a dataset collected during the first few years of the Seychelles warbler study found that (a) nests with helpers received a higher amount of total provisioning compared to nests without helpers; (b) the provisioning effort of dominant females was independent of helper presence; and, (c) dominant males reduced their provisioning rates in groups with more helpers (Komdeur, 1994). Here, we replicate this study using a much larger dataset, and, for the first time in this species, disentangle the impact of help from the effects of group size (including helpers and nonhelpers) and food availability.

## 2 METHODS

### 2.1 Study population

The Seychelles warbler population on Cousin Island (29 ha; 04°20′S, 55°40′E) has been monitored closely since the mid‐1980s (Komdeur, Burke, Dugdale, & Richardson, 2016). The main breeding season is July–September, and a smaller breeding season occurs January–March (Komdeur, 1996). From 1997 onwards, ca. 96% of the population has been color‐ringed, using a unique combination of a metal British Trust for Ornithology ring and color rings (Richardson, Jury, Blaakmeer, Komdeur, & Burke, 2001). We recorded the identity of all color‐ringed birds present in each territory, and the sex of all birds has been molecularly determined since 1993 using blood samples (Griffiths, Double, Orr, & Dawson, 1998). Dominant birds, defined as the pair‐bonded male and female in a territory based on their behavioral interactions and nesting behavior (Richardson, Burke, & Komdeur, 2002), form long‐term pair bonds. Groups may contain 0–5 sexually mature (>5 months old) subordinates, which are usually retained offspring (Groenewoud et al., 2018; Kingma et al., 2016; Richardson et al., 2002) and typically produce one clutch per season of a single egg (87%; range 1–3 eggs). Nestlings fledge 18–20 days after hatching and become independent around 88 days of age (Komdeur, 1991). Subordinate birds were defined as “helpers” when they were observed brooding or provisioning offspring at least once during a nest watch, with assessments made at every nest watch. Territories were checked for breeding activity at least once every 2 weeks by following the dominant female for a minimum of 15 min. Once breeding, focal territories were checked every week for at least 15 min to determine nest building, brooding or feeding activity.

### 2.2 Provisioning observations

We measured nestling and fledgling provisioning rates at nests produced between 1996 and 2015. Provisioning watches with >10% of provisioning events by unidentified birds were excluded from the analyses (*N = *178 of 701 watches), with further nest watches excluded with no monthly insect abundance estimate (*N = *74). A total of 449 nest watches were included in our analyses, measuring 60–90 min each. These watches included a total of 889 dominant breeder provisioning watches (Supporting Information, Table S1) over 353 nests, attempted by 349 unique male–female pairs. The total number of unique birds included 214 dominant females and 209 dominant males. For three and six out of 449 nest watches, no dominant female or male respectively was observed provisioning, resulting in a total of 889 dominant breeder provisioning watches (Supporting Information Table S1). Each nest was watched for a mean of 1.3 times (95% CI = 1.2–1.3), with a mean total observation duration per nest of 82 min (95% CI = 79–86; range = 60–185 min). Of these 449 nest watches, 45% included helpers and 36% included subordinate nonhelpers (Supporting Information Table S2). Ninety nests were watched more than once, and 12 (13%) of these had a subordinate that was classified as a helper in one watch and a nonhelper in another watch. We scored helping on a per nest watch basis, as we were interested in how the behavior of the dominants varied in relation to the number of subordinates that were currently helping with provisioning or brooding.

Provisioning rates were calculated as the number of nest visits during which the nestling(s) was fed. Sex‐specific parental investment, including building and guarding the nest or brooding, is known to change over the course of the pre‐ and posthatching stages (Komdeur & Kats, 1999). To account for different types of observations as a proxy of chick developmental state, we grouped provisioning watches into three categories: (a) provisioning and brooding: a nestling was fed in the nest and a female was still brooding; (b) provisioning nestling: a nestling was fed in the nest and no brooding occurred; and, (c) provisioning fledgling: a fledgling was fed away from the nest. Although brooding during provisioning can occur as a way to protect the nestling from the environment, most brooding occurred immediately after hatching (field observations).

### 2.3 Monthly insect abundance and territory quality

Seychelles warblers are insectivorous, taking 98% of their insect food from the undersides of leaves (Komdeur, 2006; Komdeur & Pels, 2005). The number of insects present in a territory is a useful index of territory quality (Komdeur, 1994) which reflects the number of fledglings, independent offspring and yearlings produced (Komdeur & Pels, 2005). Insect abundance was estimated by counting the number of insects on the undersides of 50 leaves of the most abundant plant species (Eikenaar, Richardson, Komdeur, & Brouwer, 2010; Komdeur, 1991), at 15 (until 1999) or 14 (after 1999) fixed locations on the island once every month. Monthly insect abundance was calculated as the mean insect abundance across these locations, with insect abundances in each territory extrapolated from the nearest insect count location (Komdeur, 1991). Furthermore, to provide an overall index of territory quality for each territory and investigate long‐term effects of environment on investment, we calculated mean standardized territory quality per territory over all seasons (Hammers, Richardson, Burke, & Komdeur, 2012)*. *These estimates were calculated as insect abundance per unit leaf area (dm^2^) multiplied by vegetation abundance score, multiplied by territory size. Leaf area was estimated in 1991 by measuring the area of five leaves of each abundant plant species at 50 random sites on the island (Komdeur, 1991). Vegetation abundance was scored each season by determining the presence of all plant species at 20 random points in a territory in the following height bands: 0–0.75 m, 0.75–2 m, 2–4 m, and at 2 m intervals thereafter (Komdeur, 1991). Territory sizes were measured each season using ArcGIS 9; territory boundaries were based on observations of individual warblers and the outcomes of territory disputes. Territory quality estimates were standardized across territories in each breeding season, by mean centering and dividing by two standard deviations (Gelman & Hill, 2007).

### 2.4 Statistical methods

We performed generalized linear mixed model analyses in MCMCglmm 2.24 (Hadfield, 2010), which takes a Bayesian approach, in R 3.4.0 (R Core Team, 2017). We first investigated the impact of helper care on the dominants’ parental investment by modeling the number of provisioning visits by each dominant individual to offspring. Along with the number of helpers, we included the sex of the dominant individual, number of offspring, group size, provisioning watch type (provisioning and brooding, provisioning nestling, provisioning fledgling), monthly insect abundance and territory quality index as fixed effects. To explore sex differences in provisioning in response to helper presence or type of provisioning watch (a proxy for chick developmental state), we tested for an interaction between the number of helpers and sex of the dominant individual, and provisioning watch type and sex of the dominant individual. To account for varying observation duration, yet retain variation, the log of the watch duration was also included in the fixed structure (log was applied due to right skew) and a prior was specified to set its regression coefficient to 1 (i.e., observation duration was treated as an offset). To control for repeated measures from dominant individuals that provisioned in more than one breeding season, we included bird identity as a random effect, using an idh variance structure (heterogeneous error variance with no covariance) to allow sex‐specific variances to be estimated. To control for multiple provisioning watches and simultaneous watches of males and females at the same nest, we included the random effects of provisioning watch identity nested within nest identity. We did not include territory identity as the posterior density plot of territory identity was poor. Multiple provisioning records from the same territory could therefore be a problem in our analyses, so to best control for this without including territory identity, we included individual identity and territory quality, to account for multiple records from the same birds and birds in similar quality territories potentially having similar provisioning rates. To control for differences between observers we included observer identity as a random effect. For the random effects, we applied parameter expanded priors (noncentral scaled *F*‐distribution; *V* = 1, nu = 0.002, alpha.mu = 0, alpha.V = 1,000) to aid chain mixing, as the variance was close to zero and inverse‐Wishart distributed priors have high density at values close to zero (Hadfield, 2015). For bird identity and residual variance, the expanded prior was structured as a 2×2 matrix to estimate variances for dominant males and females separately. The model had a Poisson error distribution and log link, was run for 4.5 × 10^5^ iterations with a burn‐in of 5 × 10^4^ and thinning of 400.

To test whether helper effects were additive or compensatory, we modeled the total number of provisioning visits per watch (i.e., by all dominants and helpers combined). This model was the same as the provisioning model except that the response was the total number of feeds, the parameters describing sex and bird identity were omitted and the model was run for 2.1 × 10^7^ iterations with a 1 × 10^6^ burn‐in and 2 × 10^3^ thinning. Provisioning observations of nests with more than one nestling can be confounded by factors such as sibling competition (Bebbington et al., 2017) and reduced statistical power resulting from low sample size of nests with more than one nestling (48/523). We therefore ran additional models with identical settings on single nestling nests thereby excluding the number of offspring as a fixed effect (Supporting Information) to confirm that this did not alter our conclusions.

To assess model convergence, we checked that the: (a) autocorrelation for all parameters was <0.1; (b) variance estimates passed the Heidelberger and Welch's convergence diagnostic, which test if successive samples are drawn from a stationary distribution; (c) variance estimates passed the Geweke diagnostic, which tests for equality of the means of the first 10% and last 50% of the Markov chain; and (d) variance inflation between fixed effects was <3 to avoid collinearity (Cowles & Carlin, 1996; Geweke, 1991; Heidelberger & Welch, 1983). We evaluated if the 95% credibility intervals (95% CrI) of the posterior modes overlapped zero, where a departure from zero was interpreted as a significant effect.

## 3 RESULTS

**Figure 1:**
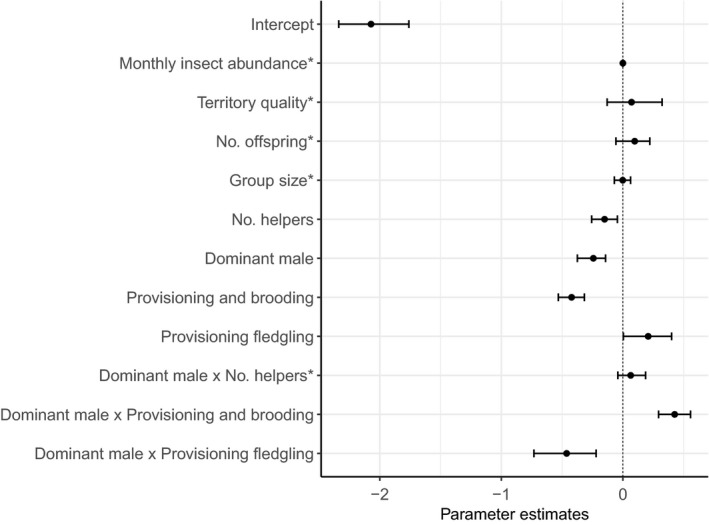
Posterior density estimates of parameter modes, and their 95% credible intervals, for the fixed effects used to model the number of feeds by dominant Seychelles warblers with or without helpers: monthly insect abundance, index of territory quality, number of offspring (1 = 808, 2 = 79, 3 = 2), group size (2 = 280, 3 = 425, 4 = 150, 5 = 30, 6 = 4), number of helpers (0 = 492, 1 = 350, 2 = 47), sex of the dominant bird (male = 446, female = 443; contrast = female), watch type (provisioning and brooding = 438, provisioning nestling = 384, provisioning fledgling = 67; contrast = provisioning nestling). *Parameters whose credible intervals do not overlap zero

**Figure 2:**
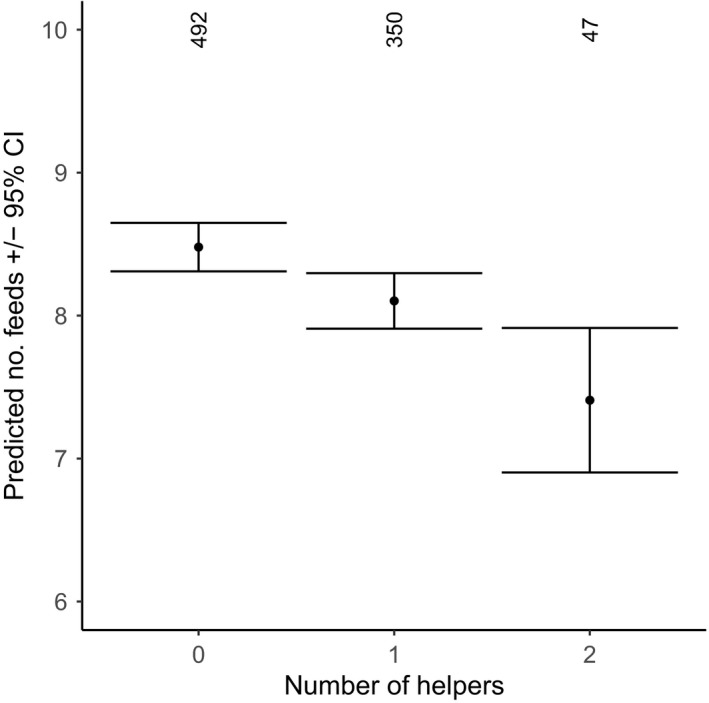
The predicted mean number of feeds in provisioning watches by dominant Seychelles warblers in respect to the number of helpers present. Error bars represent 95% confidence intervals and numbers at the top of the graph represent number of dominant breeder watches

**Figure 3:**
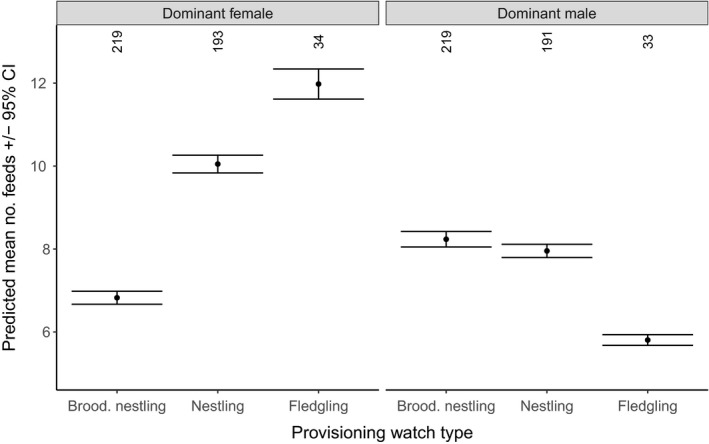
The predicted mean number of feeds during provisioning watches by dominant Seychelles warbler males and females in relation to the three types of provisioning watches: provisioning and brooding nestlings (brood. nestling), provisioning nestlings (nestling) and provisioning fledglings (fledgling). Error bars represent 95% confidence intervals and numbers at the top of the graph represent number of dominant breeder watches

Both male and female dominants showed lower provisioning effort when more helpers aided in provisioning (12.9% reduction in feeds/hour per helper, from 8.5 (no helpers, *N* = 492) to 8.1 (one helper; *N* = 350) and 7.4 feeds/hour (two helpers; *N* = 47); Figures 1 and 2). This load‐lightening effect was similar for males and females as no interaction between the sex of the dominant and the number of helpers was found (Figure 1). An interaction between the sex of the dominant and provisioning watch type revealed that the provisioning rates of dominant males were 27.0% higher to nestlings (8.0 feeds/hr) versus fledglings (5.8 feeds/hr; Figures 1 and 3). The opposite pattern was observed in dominant females, which fed fledglings almost twice as much as nestlings (12.0 vs. 6.8 feeds/hr; Figure 3). Feeding rates were not significantly related to monthly insect abundance, territory quality, number of offspring, or group size (Figure 1).

**Figure 4:**
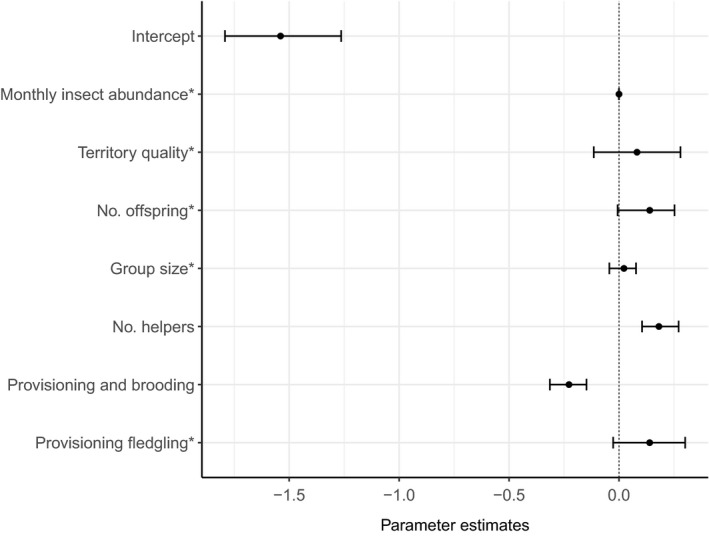
Posterior density estimates of parameter modes, and their 95% credible intervals, for the fixed effects used to model the total number of feeds received by the Seychelles warbler offspring from all feeding birds per provisioning watch: monthly insect abundance, index of territory quality, number of offspring (1 = 408, 2 = 40, 3 = 1), group size (2 = 141, 3 = 214, 4 = 77, 5 = 15, 6 = 3), number of helpers (0 = 248, 1 = 177, 2 = 24), watch type (provisioning and brooding = 222, provisioning nestling = 193, provisioning fledgling = 34; contrast =provisioning nestling). *Parameters whose credible intervals do not overlap zero

**Figure 5:**
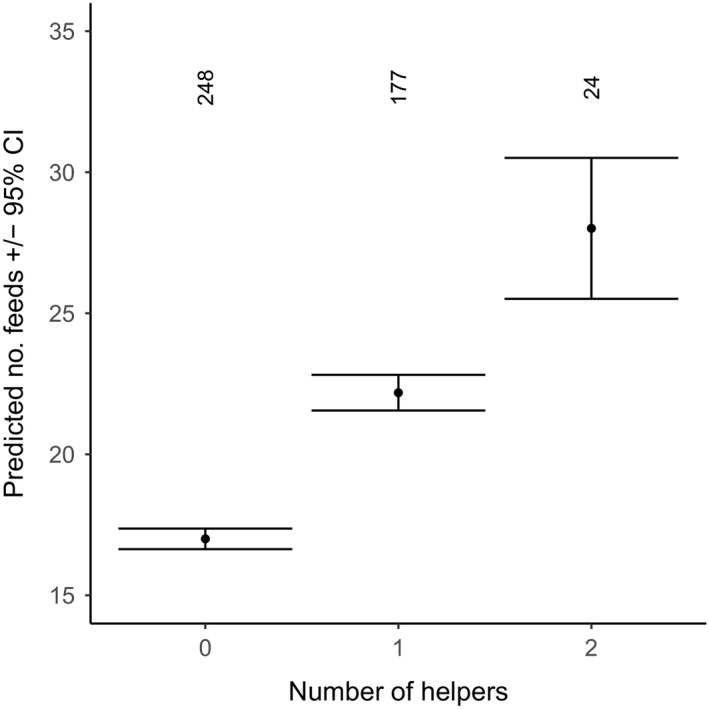
The predicted total number of feeds during provisioning watches in relation to the number of helpers present in the territory. Error bars represent 95% confidence intervals and numbers at the top of the graph represent number of dominant breeder watches

**Figure 6:**
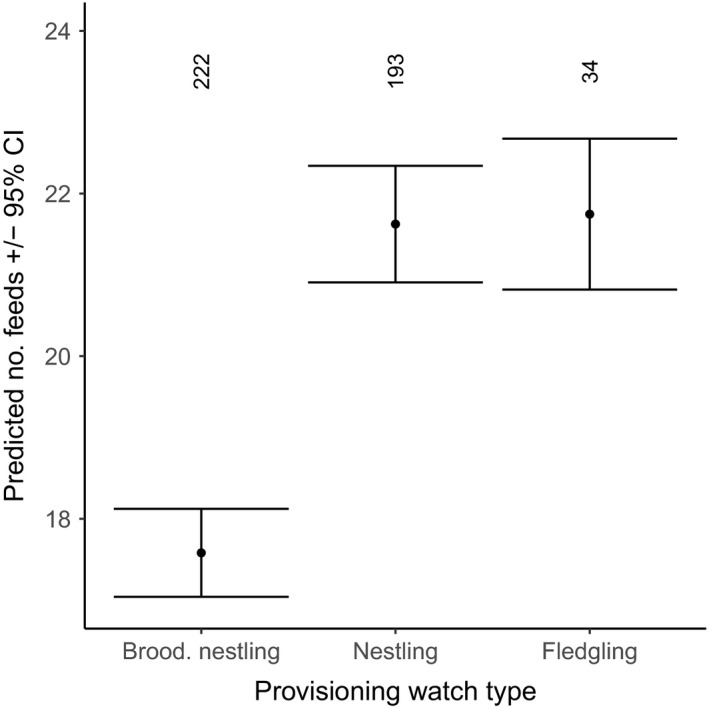
The predicted total number of feeds received by the Seychelles warbler offspring from all feeding birds in relation to the three types of provisioning watches: provisioning and brooding nestlings (brood. nestling), provisioning nestlings (nestling) and provisioning fledglings (fledgling). Error bars represent 95% confidence intervals and numbers at the top of the graph represent number of dominant breeder watches

We found an increase in total provisioning when helpers were feeding and also when more helpers were involved (Figure 4). A single helper resulted in an increase of 30.5% (22.2 visits per hour, *N* = 177, compared to 17.0 feeds in pairs, *N* = 248) provisioning visits per hour, and a second helper increased the total provisioning effect to a 64.7% increase (28.0 feed/hour, *N* = 24; Figure 5). The total number of provisioning visits each hour to nestlings also being brooded was 23.0% less than to nestlings only being provisioned (17.6 vs. 21.6; Figures 4 and 6). The total number of provisioning visits received by offspring was not correlated with group size, number of offspring, territory quality or monthly insect abundance (Figure 4). Excluding nests with more than one offspring from these models did not change the direction or significance of our results (Supporting Information). Together, these results indicate load‐lightening and total provisioning increased with additive feeding investment by helpers.

## 4 DISCUSSION

Our analyses of the long‐term Seychelles warbler dataset revealed both additive and compensatory helper effects in this species. Helpers lightened the provisioning load of dominant individuals and increased the total number of provisioning trips to the nestlings. These results were not the confounding result of group size or territory quality. Moreover, in addition to subordinates being defined as helpers if they were observed provisioning, subordinates could also be classified as helpers if they were observed aiding with the brooding only (and not provisioning). Therefore, this is a conservative analysis and the actual additive and compensatory effects might be higher. The increased total nest provisioning effort resulting from additive helper provisioning could lead to higher nestling survival (Hatchwell, 1999a; MacColl & Hatchwell, 2003; Valencia, Cruz, Carranza, & Mateos, 2006; Woxvold & Magrath, 2005). Indeed, in the Seychelles warbler, this may well explain the higher survival of offspring in their first year (Komdeur, 1994) and beyond (Brouwer et al., 2012), leading to direct fitness benefits for parents.

We demonstrated that, in addition to additive care, helpers also provide load‐lightening benefits for dominant individuals, as dominants of both sexes reduced provisioning rates when aided by helpers. In some, but not all, species (Heinsohn, 2004; Kingma et al., 2010) such load‐lightening benefits have been associated with increased survival of dominants with helpers. In the Seychelles warbler, survival of dominants with and without helpers is similar (Komdeur, 1994; Hammers et al. [Link]), except among very old dominants when those that receive help show higher survival (Hammers et al. [Link]). While it may be that load‐lightening effects on breeder survival are only obvious in some circumstances (i.e., when breeders are old), other reproductive components (like renesting opportunities or time between nesting attempts) may also be affected by breeders reducing their current workload. Future work will need to reveal whether such effects may explain selection on breeders reducing workload in response to help.

We found that provisioning rates of male dominants were lower than those of female dominants in most provisioning watches. Sex‐related differences in the parental investment of the dominants are not uncommon (Hatchwell, 1999a; MacColl & Hatchwell, 2003), and are proposed to result from diverging cost‐benefit trade‐offs between the sexes (MacColl & Hatchwell, 2003). Several studies have shown that the genetic relatedness of the carer to the brood affects investment, where male uncertainty of parentage can result in lower amounts of care (e.g., Burke, Daviest, Bruford, & Hatchwell, 1989; Neff, 2003; Kokko & Jennions, 2012). In the Seychelles warbler, male breeders are on average less related to the offspring than females, due to the 44% extra‐pair paternity occurring in this species (Hadfield, Richardson, & Burke, 2006; Richardson et al., 2001), which may explain the overall lower provisioning by breeder males.

The observation that sex‐specific investment changed over the course of the breeding event may suggest that other aspects, besides certainty of parentage, affect the symmetry of provisioning between sexes, as has been observed in other species (Cockburn et al., 2008; Meade et al., 2010). For example, females might reduce the costs of investment before the nestling period by decreasing egg size when assisted by helpers (Russell, Langmore, Gardner, & Kilner, 2008; Dixit et al., 2017; but see Koenig, Walters, & Haydock, 2009). In the Seychelles warbler, females predominantly build the nest and brood the egg, and spend less time foraging compared to males, who guard the nest (Komdeur & Kats, 1999). This, in combination with ongoing brooding of newly hatched chicks, may suggest higher costs, such as time investment, for females during the prenestling and young‐nestling period, which could explain lower provisioning effort of the dominant female compared to the dominant male shortly after hatching. Therefore, the most suitable investment strategy may change within the breeding season and fine‐scaled studies are required to understand the evolution of parental care (Savage, Browning, Manica, Russell, & Johnstone, 2017).

Our results differ from previous findings of provisioning effort in the Seychelles warbler in relation to helper presence. Komdeur (1994) found a load‐lightening effect for dominant males only when three or more helpers were present. The relatively higher degree of load lightening identified here, for both sexes and with a smaller number of helpers, could result from the higher data accuracy used in the current study, with 97% of the Cousin bird population ringed versus <50% in the previous study. Alternatively, these results could suggest that the cost‐benefit trade‐offs for dominant individuals may have changed since Komdeur's earlier Seychelles warbler study. For instance, an increase in offspring survival (e.g., due to higher quality of insects or increased protection from the environment; Komdeur & Pels, 2005) would allow parents to relax investment into the current brood.

## 5 CONCLUSION

Our study adds to the growing evidence that both compensatory and additive care can apply at the same time within one species. These simultaneous parental care strategies are fundamental to the evolutionary maintenance of cooperative behavior. The exact fitness effects of both load‐lightening and additive care, as well as sex‐specific changes in fitness benefits during the breeding season need to be explored in the future.

## CONFLICT OF INTEREST

None declared.

## Data Availability

Data is made available on Figshare https://doi.org/10.6084/m9.figshare.6977564.v1. R code is made available on GitHub: https://github.com/Seychelle-Warbler-Project/vanBoheemen_Lotte/.
