# Supplementary material for: Compensatory and additive helper effects in the cooperatively breeding Seychelles warbler (Acrocephalus sechellensis)
Source: Ecol Evol. 2019 Feb 14;9(5):2986–95. doi: 10.1002/ece3.4982 (PMC6405499; doi:10.1002/ece3.4982)
Supplement: Supplementary file 1 [file ECE3-9-2986-s001.docx]

**SUPPORTING INFORMATION TO**

**Compensatory and additive helper effects in the cooperatively breeding Seychelles warbler (*Acrocephalus sechellensis*).**

Lotte A. van Boheemen^1,2^, Martijn Hammers^2^, Sjouke A. Kingma^2,3^, David S. Richardson^4,5^, Terry Burke^6^, Jan Komdeur^2^, Hannah L. Dugdale^6,7^

Corresponding author: Lotte A. van Boheemen: la.vanboheemen@gmail.com

^1^ School of Biological Sciences, Monash University, Clayton, VIC, Australia

^2^ Behavioural and Physiological Ecology, Groningen Institute for Evolutionary Life Sciences, University of Groningen, Groningen, the Netherlands

^3^ Behavioural Ecology Group, Department of Animal Science, Wageningen University & Research, Wageningen, The Netherlands.

^4^ School of Biological Sciences, University of East Anglia, Norwich, UK

^5^ Nature Seychelles, Mahé, Republic of Seychelles

^6^ Department of Animal and Plant Sciences, University of Sheffield, Sheffield, UK

^7^ Faculty of Biological Sciences, School of Biology, University of Leeds, Leeds, UK

This supplementary information contains:

1. Two summary tables of the nest watch dataset
2. Supplementary methods and results, where a subset of nests containing single nestlings are analysed to check that the results are not confounded by sibling competition, along with 6 supplementary figures displaying these results.

**Table S1.** Number of *Acrocephalus sechellensis* nest watches each year for each dominant breeder per watch type. For 3 and 6 out of 449 nest watches no dominant female or male respectively was observed provisioning, resulting in a total of 889 dominant breeder provisioning watches.

|  | **Dominant female** | | | | **Dominant male** | | | |  |
| --- | --- | --- | --- | --- | --- | --- | --- | --- | --- |
| **Year** | **Brooding nestling** | **Nestling** | **Fledging** | **Total** | **Brooding nestling** | **Nestling** | **Fledging** | **Total** | **Grand Total** |
| 1996 | 4 | 6 |  | 10 | 4 | 6 |  | 10 | 20 |
| 1997 | 15 | 4 | 2 | 21 | 15 | 4 | 2 | 21 | 42 |
| 1998 | 29 | 10 |  | 39 | 28 | 10 |  | 38 | 77 |
| 1999 | 17 | 15 |  | 32 | 17 | 15 |  | 32 | 64 |
| 2003 | 14 | 11 |  | 25 | 14 | 11 |  | 25 | 50 |
| 2004 | 9 | 4 |  | 13 | 9 | 4 |  | 13 | 26 |
| 2005 | 9 | 5 |  | 14 | 9 | 5 |  | 14 | 28 |
| 2006 | 16 | 13 |  | 29 | 16 | 12 |  | 28 | 57 |
| 2007 | 1 | 2 | 1 | 4 | 1 | 2 | 1 | 4 | 8 |
| 2008 | 7 | 2 | 2 | 11 | 7 | 2 | 2 | 11 | 22 |
| 2009 | 3 | 2 | 4 | 9 | 4 | 2 | 4 | 10 | 19 |
| 2010 | 18 | 17 | 1 | 36 | 18 | 17 | 1 | 36 | 72 |
| 2011 | 7 | 15 |  | 22 | 6 | 15 |  | 21 | 43 |
| 2012 | 8 | 7 | 8 | 23 | 9 | 6 | 7 | 22 | 45 |
| 2013 | 8 | 11 | 7 | 26 | 8 | 11 | 7 | 26 | 52 |
| 2014 | 33 | 45 | 9 | 87 | 33 | 45 | 9 | 87 | 174 |
| 2015 | 21 | 24 |  | 45 | 21 | 24 |  | 45 | 90 |
| **Grand Total** | 219 | 193 | 34 | 446 | 219 | 191 | 33 | 443 | 889 |

**Table S2.** Number of *Acrocephalus sechellensis* nest watches for which helpers and/or non-helpers were present.

|  |  | **Number of non-helpers** | | | | |
| --- | --- | --- | --- | --- | --- | --- |
|  |  | **0** | **1** | **2** | **3** | **Grand Total** |
| **Number of helpers** | **0** | 158 | 71 | 16 | 3 | 248 |
|  | **1** | 115 | 50 | 10 | 2 | 177 |
|  | **2** | 16 | 7 | 1 |  | 24 |
|  | **Grand Total** | 289 | 128 | 27 | 5 | 449 |

***Analyses on data subsets with single offspring nests***

Provisioning observations of nests with more than one offspring can be confounded by factors such as sibling competition (Bebbington *et al.* 2017) and reduced statistical power resulting from low sample size of provisioning observations of such nests (48/523). We therefore ran additional models with identical settings, excluding the number of offspring as a fixed effect. Excluding the number of offspring from these models did not change the direction or significance of our results, as shown below.

Both male and female dominants showed lower provisioning effort when more helpers aided in provisioning (13.3% reduction in predicted provisioning rates per helper, from 8.3 (no helpers; N = 458) to 8.1 (one helper; N = 309) and 7.2 feeds/hour (two helpers; N = 41); Fig. S2). This load-lightening effect was similar for males and females as the 95% credible intervals of the interaction between the sex of the dominant and the number of helpers included zero (Fig. S1). An interaction between the sex of the dominant and the type of watch (brooded nestlings, nestlings, fledglings) revealed that the provisioning rates of dominant males were 32.2% higher to nestlings (8.2 feeds/hour) compared to fledglings (5.6 feeds/hour; Fig. S1 & S3). The opposite pattern was observed in dominant females, which fed fledglings almost twice as much more than nestlings (11.9 versus 6.7 feeds/hour; Fig. S3). Feeding rates were not significantly related to monthly insect abundance, territory quality, number of offspring, or group size (Fig. S1).

We found a strong increase in total provisioning rate when more helpers were feeding (Fig. S4) when we only analysed provisioning watches with only one offspring. A single helper resulted in an increase of 25.7% provisioning visits per hour (21.0, *N* = 156, compared to 16.7 feeds in pairs, *N* = 231, and a second helper in an increase of 29.0% (27.1 feeds/hour, *N* = 21; Fig. S5). The total number of provisioning visits per hour to nestlings being brooded was 17.1% less than for nestlings only being provisioned (17.4 versus 21.0 feeds/hour; Fig. S4 & S6). The total provisioning visits received by the offspring was not correlated with group size, territory quality or monthly insect abundance (Fig. S4). Together, these results indicate load-lightening and total provisioning increased with additive feeding investment by helpers.

**Figure S1** Posterior density estimates of parameter modes, and their 95% credible intervals, for the fixed effects used to model the number of feeds by dominant Seychelles warblers with one offspring with or without helpers: monthly insect abundance, index of territory quality, group size (2-6), number of helpers (0-2), sex of the dominant bird (contrast = female), watch type (provisioning and brooding, provisioning nestling, provisioning fledgling; contrast = provisioning nestling). * indicates parameters whose credible intervals do not overlap zero.

**Figure S2** The mean number of feeds by dominant birds in relation to number of helpers present at provisioning watches with one offspring only. Error bars represent 95% confidence intervals and numbers at the top of the graph represent number of nests.

**Figure S3** The mean number of feeds by dominant male and female Seychelles warblers, in provisioning watches with one offspring only, in relation to the three types of provisioning watches: provisioning & brooding nestlings (brood. nestling), provisioning nestlings (nestling) and provisioning fledglings (fledgling). Error bars represent 95% confidence intervals and numbers at the top of the graph represent sample sizes.

**Figure S4** Posterior density estimates of parameter modes, and their 95% credible intervals, for the fixed effects used to model the total number of feeds per provisioning watch with one offspring only: monthly insect abundance, index of territory quality, number of offspring (1-3), group size (2-6), number of helpers (0-2), watch type (provisioning and brooding, provisioning nestling, provisioning fledgling; contrast = provisioning nestling). * indicates parameters of which the credible intervals do not overlap zero.

**Figure S5** The total number of feeds during provisioning watches in relation to the number of helpers present for nests with one offspring only. Error bars represent 95% confidence intervals. Numbers at the top of the graph represent number of nests.

**Figure S6** The total number of feeds received, at provisioning watches with one offspring only, in relation to the three types of provisioning watches: provisioning & brooding nestlings (brood. nestling), provisioning nestlings (nestling) and provisioning fledglings (fledgling). Error bars represent 95% confidence intervals. Numbers at the top of the graph represent sample sizes.
